# 5-HT_1A _gene promoter polymorphism and [^18^F]MPPF binding potential in healthy subjects: a PET study

**DOI:** 10.1186/1744-9081-6-37

**Published:** 2010-07-07

**Authors:** Amélie Lothe, Claudette Boni, Nicolas Costes, Sandrine Bouvard, Philip Gorwood, Franck Lavenne, Marion Alvarez, Philippe Ryvlin

**Affiliations:** 1 CTRS-IDEE, Lyon, France; 2 INSERM U821, University Claude Bernard Lyon 1, INFL, Lyon, France; 3 INSERM U675, Faculté Xavier Bichat, University Paris VII, Paris, France; 4 CERMEP imagerie du vivant, Lyon, France; 5 Department of Functional Neurology and Epileptology, Hospices Civils de Lyon, France

## Abstract

**Background:**

Previous Positron Emission Tomography (PET) studies of 5-HT_1A _receptors have shown an influence of several genetic factors, including the triallelic serotonin transporter gene-linked polymorphic region on the binding potential (BP_ND_) of these receptors. The aim of our study was to investigate the relationship between a 5-HT_1A _promoter polymorphism and the binding potential of another selective 5-HT_1A _receptor antagonist, [^18^F]MPPF, in healthy subjects.

**Methods:**

Thirty-five volunteers, including 23 women, underwent an [^18^F]MPPF scan and were genotyped for both the C(-1019)G 5-HT_1A _promoter polymorphism and the triallelic serotonin transporter gene-linked polymorphic region. We used a simplified reference tissue model to generate parametric images of BP_ND_. Whole brain Statistical Parametric Mapping and raphe nuclei region of interest analyses were performed to look for an association of [^18^F]MPPF BP_ND _with the C(-1019)G 5-HT_1A _promoter polymorphism.

**Results:**

Among the 35 subjects, 5-HT_1A _promoter genotypes occurred with the following frequencies: three G/G, twenty-one G/C, and eleven C/C. No difference of [^18^F]MPPF BP_ND _between groups was observed, except for two women who were homozygote carriers for the G allele and showed greater binding potential compared to other age-matched women over the frontal and temporal neocortex. However, the biological relevance of this result remains uncertain due to the very small number of subjects with a G/G genotype. These findings were not modified by excluding individuals carrying the S/S genotype of the serotonin transporter gene-linked polymorphic region.

**Conclusions:**

We failed to observe an association between the C(-1019)G 5-HT_1A _promoter polymorphism and [^18^F]MPPF binding in healthy subjects. However our data suggest that the small number of women homozygote for the G allele might have greater [^18^F]MPPF BP_ND _relative to other individuals. This finding should be confirmed in a larger sample.

## Background

The binding potential (BP) of [^11^C]WAY100635 at 5-HT_1A _receptors is reported to be influenced by several genetic factors, including the serotonin transporter gene-linked polymorphic region (5-HTTLPR) [1,2]. We have recently evaluated the impact of this polymorphism on the BP of another selective 5-HT_1A _receptor antagonist, [^18^F]MPPF (4-(2'-methoxyphenyl)-1-[2'-(N-2-pirydynyl)-p-fluorobenzamido]-ethyl-piperazine), given that [^11^C]WAY100635 and [^18^F]MPPF demonstrate several functional differences [3]. [^18^F]MPPF is characterized by an affinity for 5-HT_1A _receptors in rat hippocampal membrane homogenates (Ki = 3.3 nM) which is lower than that for [^11^C]WAY100635 (Ki = 0.8 nM) [4], and more comparable to that of endogenous serotonin (5-HT) (Ki = 4.17 nM) [5]. Furthermore, in contrast to [^11^C]WAY100635, [^18^F]MPPF is reported to be sensitive *in vivo *to the concentration of extracellular 5-HT [6-8]. Accordingly, we previously observed that healthy women homozygote carriers of the S allele of 5-HTTLPR had significantly greater [^18^F]MPPF BP than carriers of at least one L_A _allele [3].

In the present study, we have extended our research to examine a possible association between C(-1019)G 5-HT_1A _promoter polymorphism and [^18^F]MPPF BP. This polymorphism is located in the regulatory region of the 5-HT_1A _promoter which inhibits transcriptional repression of the 5-HT_1A _gene [9,10], and is part of a 26 base pair imperfect palindrome [11]. This palindromic region is recognized by two transcription factors; the nuclear deformed epidermal autoregulatory factor (NUDR, Deaf-1) and Hes5. This recognition occurs in an allele-specific manner, such that these proteins bind to the C allele but not the G allele. The G allele is reported to abolish repression by NUDR in raphe but not hippocampal neuron cultures [11]. Thus, the G allele is supposedly associated with higher expression of 5-HT_1A _receptors in the raphe nuclei and decreased 5-HT release, consistent with the association reported between G/G genotype and major depression [11,12].

The association of C(-1019)G 5-HT_1A _promoter polymorphism and 5-HT_1A _receptor binding has been studied in humans in three [^11^C]WAY-100 635 PET studies [1,12,13]. One of these studies was performed using a homogenous group of healthy subjects and failed to detect any significant relationship between C(-1019)G 5-HT_1A _promoter polymorphism and [^11^C]WAY-100635 BP [1]. The other two studies, performed using a mixed population of depressed and healthy individuals, demonstrated greater BP in the raphe nuclei, the amygdala, and the hippocampus in carriers with at least one G allele compared to the C/C genotype [12,13].

In the present study, we sought to identify possible associations between C(-1019)G 5-HT_1A _promoter polymorphism and [^18^F]MPPF BP in a homogenous group of healthy subjects, and further examined the potential confounding impact of triallelic 5-HTTLPR polymorphism.

## Methods

Thirty-five healthy subjects were selected on the basis of (i) no sign or history of neurological, psychiatric, cardiovascular, pleuro-pulmonary or haematological illness, (ii) no ongoing central nervous system (CNS)-active treatment including neuroleptic, antiparkinsonian, methyl-dopa, β-blocker, monoamine oxidase inhibitor-A or -B, tricyclic antidepressant, thymoregulator or antimigraine treatment, (iii) no hormone replacement therapy, and (iv) a score below the threshold for depression (< 7) for the GHQ-28. All subjects gave their informed consent to participate in this study, which was approved by the local Ethics committee (CCPPRB, Centre Léon Bérard, Lyon) in accordance with the Declaration of Helsinki and French regulations on Biomedical Research.

Genomic DNA was extracted from endobuccal cell swabs, according to the BuccalAmp DNA Extraction Kit from Epicentre. Allele-specific polymerase chain reaction (PCR) amplification was performed according to the protocol originally described by Hong et al. [14], and enabled us to classify each subject into one of the three following groups: C/C, C/G or G/G. In addition, all subjects were genotyped for the triallelic 5-HTTLPR polymorphism and classified as S/S, S/L_A _and L_A_/L_A _(as reported previously; see [3] for details).

All subjects also underwent a 3-D anatomical T1-weighted sequence on a 1.5-T Siemens Magnetom scanner (Siemens AG, Erlangen, Germany). Data were acquired in the sagittal plane with 1 mm^3 ^voxels and an anatomical volume that covered the whole brain. Magnetic resonance images (MRIs) were visually analysed by a neurologist to ensure that no brain lesion or malformation was present.

The methodology of tracer production, scan acquisition and data pre-processing was the same as previously described [15]. To summarize, [^18^F]MPPF was acquired with a CTI Exact HR+ scanner for 60 min after the injection of 186 ± 24 MBq of [^18^F]MPPF. Specific activity ranged between 37 and 111 GBq/μmol [16]. Sinograms were normalized, attenuated, scatter corrected and then reconstructed with filtered backprojection (Hanning filter: cutoff, 0.5 cycle/voxel). This yielded a dynamic set of 35 volumes of 128 × 128 × 63 voxels with a voxel size of 2.04 × 2.04 × 2.42 mm^3^. All PET acquisitions were performed during the afternoon, and the patient's level of vigilance was supervised throughout the entire PET acquisition to avoid somnolence or sleep.

Parametric images of binding potential (BP_ND _[non displaceable], according to consensus nomenclature [17]) values were obtained using a simplified reference tissue model (SRTM) [18] previously validated by our group for [^18^F]MPPF studies [19]. Cerebellar white matter, which is assumed to be devoid of 5-HT_1A _specific binding [20], was used as a reference region [19]. Parametric images of BP_ND _were transformed into a standard stereotaxic space (MNI template of the ICBM Project, [21]) using Statistical Parametric Mapping (SPM2, Wellcome Trust Centre for Neuroimaging, UCL, London, UK; http://www.fil.ion.ucl.ac.uk/spm/). Normalized BP_ND _images were then smoothed using an 8 × 8 × 8 mm FWHM isotropic Gaussian kernel [22].

The BP_ND _global values were extracted for all subjects using SPM and were compared among C/C, C/G and G/G subgroups using an ANOVA and the Statistical Program for the Social Sciences (SPSS, version 12). The significance threshold was set at p < 0.05.

We searched for possible statistical differences between BP_ND _images according to C(-1019)G 5-HT_1A _promoter functional polymorphism using the linear model at each and every voxel [23]. Given that all subjects are healthy volunteers, we hypothesize an invariant residual variance between groups. All analyses were performed using SPM2 on the normalized smoothed parametric BP_ND _images. We chose an "ANCOVA by condition" design matrix, an explicit mask that includes the whole brain and brainstem, and uses global BP as a co-variable of no interest.

To further address the potential confounding role of gender, we performed subanalyses of the 23 women and 12 men, separately. Age was considered as a co-variable of no interest. We retained clusters with a threshold of p < 0.001 uncorrected at the voxel level, and a corrected threshold of p < 0.05 at the cluster level after SPM standard correction for multiple comparisons.

We further analysed [^18^F]MPPF BP_ND _in the dorsal and median raphe nuclei using a region of interest (ROI) delineated on the average [^18^F]MPPF BP_ND _image obtained from the normalized scans of our subjects by setting the threshold activity at 90% of the local maximum in the brainstem. The ROI was then displayed on the MRI template to verify its proper location in the peri-aqueducal grey matter of the brainstem. This ROI extended on the 13 consecutive slices displaying the raphe, with a total volume of 1400 mm^3^. For each subject, ROIs were then applied on normalized and smoothed parametric BP_ND _images to extract a mean BP_ND _value for this ROI. The mean BP_ND _values of raphe were compared among C/C, C/G and G/G subgroups in the total population, as well as in women and men separately, using an ANOVA and SPSS (version 12). The global BP_ND _values and age were considered as co-variables of no-interest. The significance threshold was set at p < 0.05.

In order to rule out that our findings were influenced by the previously demonstrated association between 5-HTTLPR polymorphism and [^18^F]MPPF BP_ND _[3], we reprocessed all above analyses after excluding the eight subjects who were homozygote carriers of the S allele (four men and four women).

## Results

All of our 35 healthy subjects (12 men and 23 women) were Caucasian. The allele frequencies of C(-1019)G 5-HT_1A _promoter polymorphisms were comparable to those of others studies with European subjects; 38.6% with the G allele, 61.4% with the C allele, and three individuals (8.57%) homozygous for the G allele [11,13]. Accordingly, the genotype distribution (see table 1) was in Hardy-Weinberg equilibrium (χ^2 ^= 2.48; df = 1, p = 0.11). Regarding 5-HTTLPR polymorphism, homozygote carriers of the S allele were distributed as follows: two among the C/C group, six among the C/G group and none among the G/G group.

**Table 1:** C(-1019)G 5-HT_1A _promoter polymorphism distribution.

C(-1019)G 5-HT_1A _promoter polymorphism	C/C	C/G	G/G	Total
**Number of subjects (% of population)**	11 (31.4%)	21 (60%)	3 (8.6%)	35 (100%)
**% of women**	54.5%	71.43%	66.7%	65.7%
**Mean age ± SD**	43.9 ± 14.4	44.1 ± 12.6	43.3 ± 10	44 ± 12.7
**Number of subjects with S/S genotype (%)**	2 (18.2%)	6 (28.6%)	0	8 (22.9%)

There was no difference in the mean global BP_ND _between the various allelic subgroups (C/C group: mean ± SD = 0.52 ± 0.09; C/G group: mean ± SD = 0.58 ± 0.17; G/G group: mean ± SD = 0.48 ± 0.13; p = 0.7), nor any significant influence of gender (p = 0.1).

### SPM analyses

When considering the whole sample of 35 subjects or the subgroup of 12 men, no significant difference in [^18^F]MPPF BP_ND _was observed between the three genotypes of C(-1019)G 5-HT_1A _promoter polymorphism (C/C, C/G, G/G).

**Figure 1:**
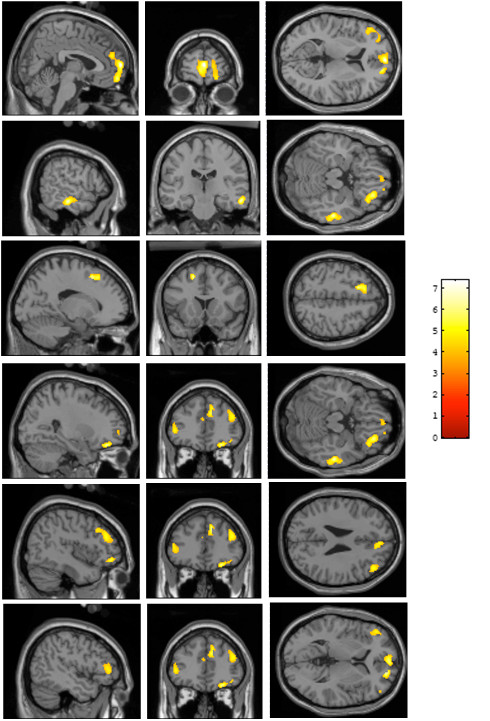
**SPM analysis of 23 women demonstrating differences between G/G genotype and C/G and C/C genotypes**. Women with G/G genotype demonstrated greater [^18^F]MPPF BP_ND _than women with C/G and C/C genotypes in the right and left mesial frontal pole, the right second and third temporal cortex (T2, T3), the left superior and middle frontal gyri (F1 and F2), the right orbitofrontal cortex, the right dorsolateral frontal cortex and the left inferior frontal gyrus (F3). All displayed regions had a height threshold at p_corrected _< 0.05 at the cluster level after correction for multiple comparisons. Colour scale: Z score.

In contrast, when the analysis was restricted to the subgroup of 23 women, we found significantly greater regional [^18^F]MPPF BP_ND _in homozygous carriers for the G allele compared to C/C and C/G individuals, while no difference was observed between these two latter populations. Differences between G/G and C/C subjects were observed in the anterior portion of the left superior and middle frontal gyri (p_corrected _= 0.042) as well as over the right and left orbitofrontal cortex (p_corrected _= 0.001) (see table 2), whereas differences between G/G and C/G individuals were found in the right and left mesial frontal pole (p_corrected _< 0.001), the anterior aspect of the right middle and inferior temporal gyri (p_corrected _= 0.004), the right orbitofrontal cortex (p_corrected _= 0.014) and the right frontal dorsolateral cortex (p_corrected _= 0.007) (see additional files 1 and 2). The same significant clusters were observed when women with C/G and C/C genotypes were combined and compared with the G/G group (see figure 1 and table 2).

**Table 2:** SPM analyses performed in the subgroup of 23 women. k is the cluster size expressed in voxels.

	23 women		
**contrast**	**anatomic brain regions covered**	**MNI coordinates (x, y, z (mm))**	**Z score, k, p**

			
**GG-CC**	left anterior superior and middle frontal gyri (F1 and F2)	-16; 16; 52	Z = 4.48, k = 239, p = 0.042*
	right and left orbitofrontal cortex	22; 58; -12	Z = 4.31, k = 550, p = 0.001*
			
**GG-CG**	right and left mesial frontal pole	4; 62; 8	Z = 5.04, k = 1884, p < 0.001*
	right anterior second and third temporal cortex (T2, T3)	60; -22; -18	Z = 4.61, k = 416, p = 0.004*
	right orbitofrontal cortex	28; 40; -18	Z = 4.48, k = 313, p = 0.014*
	right dorsolateral frontal cortex	44; 38; 26	Z = 3.94, k = 365, p = 0.007*
			
**CC-GG**	NS		
**CC-CG**	NS		
**CG-CC**	NS		
**CG-GG**	NS		
			
**GG-(CG+CC)**	right and left mesial frontal pole	4; 62; 8	Z = 4.79, k = 1407, p < 0.001*
	right anterior second and third temporal cortex (T2, T3)	60; -22; -18	Z = 4.52, k = 283, p = 0.022*
	left anterior superior and middle frontal gyri (F1 and F2)	-16; 16; 52	Z = 4.35, k = 253, p = 0.034*
	right orbitofrontal cortex	28; 40; -18	Z = 4.29, k = 298, p = 0.018*
	right dorsolateral frontal cortex	44; 38; 26	Z = 3.92, k = 324, p = 0.012*
	left anterior inferior frontal gyrus (F3)	-46; 40; 4	Z = 3.74, k = 312, p = 0.015*
			
**(CG+CC)-GG**	NS		

NS: no significant. Only significant findings are shown.

**Figure 2:**
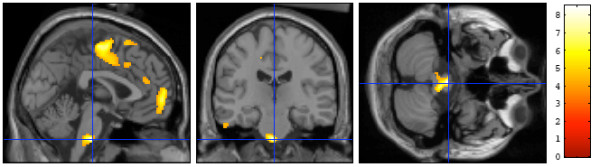
**SPM analysis performed after exclusion of homozygote carriers for the S allele of the 5-HTTLPR polymorphism**. Of the subgroup of 19 women analysed, a comparison of the G/G and C/C genotypes demonstrated comparable findings to those illustrated in figure one, with an additional significant cluster over the bulbar serotoninergic nuclei. Colour scale: Z score.

Reprocessing the above analyses after exclusion of the eight homozygote carriers for the S allele of the 5-HTTLPR polymorphism only slightly modified our findings. Indeed, only the subgroup of 19 women demonstrated significant differences in [^18^F]MPPF BP_ND _in relation to C(-1019)G 5-HT_1A _promoter polymorphism, with greater BP_ND _for those with G/G compared to those with C/C and C/G (see table 3). Significant clusters affected the same brain regions as those described above, but were more extensive and included the bulbar serotoninergic nuclei (p_corrected _= 0.021) (see figure 2).

**Table 3:** SPM analyses performed in the subgroup of 19 women after the exclusion of the homozygote carriers of the S allele of the 5-HTTLPR polymorphism.

	19 women		
**contrast**	**Anatomic brain regions covered**	**MNI coordinates (x, y, z (mm))**	**Z score, k, p**

**GG-CC**	bulbar serotoninergic nuclei	0; -22; -46	Z = 4.75, k = 244, p = 0.021*
	left superior and middle frontal gyri (F1 and F2)	-16; 18; 50	Z = 4.73, k = 480, p = 0.001*
	right mesial frontal and left supplementary motor area	2; -14; 58	Z = 4.55, k = 885, p < 0.001*
	left and right mesial frontal pole	-2; 60; -2	Z = 4.52, k = 646, p < 0.001*
	left lateral middle temporal cortex (T2)	-58; -14; -26	Z = 4.20, k = 198, p = 0.048*
	right anterior mesial frontal	12; 36; 38	Z = 4.12, k = 247, p = 0.020*
	left anterior inferior frontal gyrus (F3)	-46; 42; 6	Z = 3.66, k = 298, p = 0.009*
			
**GG-CG**	right and left mesial frontal pole	4; 62; 10	Z = 5.14, k = 3248, p < 0.001*
	right lateral orbitofrontal cortex	26; -38; -20	Z = 4.95, k = 357, p = 0.003*
	right parahippocampal gyrus	38; -16; -26	Z = 4.87, k = 273, p = 0.013*
	right temporal pole	30; 14; -38	Z = 4.75, k = 574, p < 0.001*
	right lateral second and third temporal cortex (T2, T3)	60; -22; -16	Z = 4.53, k = 545, p < 0.001*
	left lateral second and third temporal cortex (T2, T3)	-60; -12; -24	Z = 4.45, k = 383, p = 0.002*
	left temporal pole	-34; 10; -40	Z = 4.16, k = 393, p = 0.002*
	right anterior superior and middle frontal gyri (F1, F2)	-14; 26; 52	Z = 4.05, k = 214, p = 0.036*
	right frontal operculum	52; 14; 6	Z = 3.97, k = 294, p = 0.009*
	left anterior inferior frontal gyrus (F3)	-42; 28; -2	Z = 3.96, k = 501, p < 0.001*
	right dorsolateral frontal cortex	42; 36; 30	Z = 3.83, k = 373, p = 0.003*
			
**CC-GG**	NS		
**CC-CG**	NS		
**CG-CC**	NS		
**CG-GG**	NS		
			
**GG - (GC+CC)**	left and right mesial frontal pole	-2; 60; -2	Z = 4.92, k = 2877, p < 0.001*
	right lateral orbitofrontal cortex	26; -38; -20	
	right temporal pole	30; 14; -38	Z = 4.56, k = 215, p = 0.035*
	left superior and middle frontal gyri (F1, F2)	-16; 20; 50	Z = 4.49, k = 411, p = 0.002*
	right lateral second and third temporal cortex (T2, T3)	60; -22; -16	Z = 4.43, k = 342, p = 0.004*
	left lateral middle temporal cortex (T2)	-58; -12; -24	Z = 4.37, k = 321, p = 0.006*
	left superior frontal gyrus (F1)	-42; 0; 42	Z = 4.22, k = 212, p = 0.037*
	right frontal operculum	54; 12; 6	Z = 3.88, k = 234, p = 0.025*
	left anterior inferior frontal gyrus (F3)	-42; 28; -2	Z = 3.87, k = 469, p = 0.001*
	right dorsolateral frontal cortex	44; 38; 26	Z = 3.82, k = 368, p = 0.003*
			
**(GC+CC) - GG**	NS		

k is the cluster size expressed in voxels. Only significant findings are shown. NS: no significant.

### ROI analysis of the dorsal and median raphe nuclei

We failed to identify an association between C(-1019)G 5-HT_1A _promoter polymorphism and [^18^F]MPPF BP_ND _in the dorsal and median raphe, even when the analysis was restricted to women, and also after excluding homozygous carriers for the S allele for 5-HTTLPR polymorphism (see table 4).

**Table 4:** Mean [^18^F]MPPF BP_ND _values of raphe nuclei.

C (-1019)G 5-HT_1A _promoter polymorphism		C/C	C/G	G/G
	**women**	0.25 ± 0.07	0.29 ± 0.13	0.33 ± 0.22
**mean BP_ND _± SD**	**men**	0.17 ± 0.03	0.22 ± 0.04	0.14
	**all**	0.21 ± 0.07	0.27 ± 0.11	0.27 ± 0.19

## Discussion

In this PET study, no clear cut association was identified between C(-1019)G 5-HT_1A _promoter functional polymorphism and [^18^F]MPPF/5-HT_1A _receptor binding. This negative finding could reflect an unsatisfactory statistical power. However it should be stressed that the primary analysis upon which this conclusion is drawn contrasted 21 subjects with C/G genotype and 11 subjects with C/C genotype, in accordance with the general empiric consensus that neuroimaging studies that include groups of 12 subjects benefit from adequate statistical power. The 9 additional subjects with C/G genotype are likely to compensate the marginally reduced statistical power related to the 11 subjects in the C/C group. Overall, we believe that the statistical power of our main negative findings, though necessarily uncertain, is fully consistent with that used in the great majority of PET studies worldwide.

The women homozygote carriers for the G allele appeared to demonstrate greater [^18^F]MPPF BP_ND _in various frontal and temporal brain regions than those carrying at least one C allele. However, the significance of this latter finding, which was shown to be independent of the previously reported association between 5-HTTLPR polymorphism and [^18^F]MPPF BP_ND _in women, remains uncertain, due to the small number of individuals with a G/G genotype. Indeed, only three of our 35 healthy subjects (9%), including two women, were homozygous for the G allele, a figure consistent with the 8-10% prevalence of this genotype in the general population. This small number of subjects carries the risk of spurious PET findings, despite the strong statistical significance of our SPM analysis. It must be stressed that there is no easy solution to this limitation. The number of controls who can be scanned in any given PET centre is constrained by ethical issues, providing little justification to study larger populations than those reported here. Conversely, mixing data from healthy controls with those from patients suffering from various psychiatric disorders, reported in previous studies [12,13], makes it impossible to identify the direct effect of genetic polymorphism from that of its associated pathologies. An alternative for a future study would be to first screen the C(-1019)G 5-HT_1A _promoter polymorphism in a sufficiently large series of normal subjects in order to select an appropriate number of subjects homozygote for the G allele who will then undertake a [^18^F]MPPF PET study.

There is still very few data regarding the functional impact of C(-1019)G 5-HT_1A _promoter polymorphism on the serotoninergic system. Direct assessment of this polymorphism in rodents is made difficult by the fact that it is not present in rat or mouse genes. However, the G(-1019) allele was shown to abolish the repression of 5-HT_1A _autoreceptor transcription mediated in the raphe nuclei by NUDR *in vitro *[11]. The resulting over-expression of 5-HT_1A _autoreceptors should lead to a reduction of 5-HT release and a possible homeostatic over-expression of post-synaptic 5-HT_1A _receptors [12]. However, not all studies support this view. In particular, the mRNA expression and protein density of 5-HT_1A _post-synaptic receptors were shown not to respond to a reduced 5-HT concentration following lesion of the raphe or administration of PCPA [24,25]. Moreover NUDR can act as a repressor or an enhancer, depending on cell type and promoter sequence [26,27]. In particular, it was found that NUDR enhances rather than represses 5-HT_1A _transcriptional activity in hippocampal and septal cells [11]. Overall, the complex organisation of gene regulation *in vivo*, as well as the possibility of compensatory mechanisms, makes it difficult to draw any firm conclusion regarding the impact of C(-1019)G 5-HT_1A _promoter polymorphism on 5-HT_1A _receptor expression in humans.

Three [^11^C]WAY-100635 PET studies have previously investigated this issue, and have reported discordant results [1,12,13]. Two series included a mixed population of depressed or bipolar patients and healthy subjects, and both found greater [^11^C]WAY-100635 BP in carriers of at least one G allele compared to the C/C genotype, over the raphe nuclei, the amygdala, and the hippocampus [12,13]. Conversely, the third series, which, like our study, investigated a homogeneous population of normal individuals, failed to detect any association between C(-1019)G 5-HT_1A _polymorphism and [^11^C]WAY-100635 BP [1]. The data from these three series also differ in terms of gender distribution, with a greater [^11^C]WAY-100635 BP reported in women in the two mixed population studies [12,13]. In our studies, gender proved to be a crucial factor regarding the genetic variables that influence [^18^F]MPPF BP_ND _which association with 5-HTTLPR and C(-1019)G 5-HT_1A _polymorphisms were found exclusively in women [3]. Similarly, another PET study demonstrated an association between monoamine oxidase A (MAO-A) polymorphism and [^11^C]WAY-100635 BP selectively in women [28]. Other preclinical and clinical reports support sexual dimorphism in serotoninergic neurotransmission (see for review [29]). In particular, women and men differ in terms of blood and cerebral 5-HT concentration [30,31], and availability of 5-HT_1A _receptors [15].

Other important methodological issues need to be considered when interpreting available PET data, including age of subjects, data analysis, and the molecular characteristics of ligands. Binding of [^11^C]WAY-100 635 and [^18^F]MPPF to 5-HT_1A _receptor is reported to significantly decline with age, especially in women [32,15,34]. We have addressed this issue by incorporating age as a co-variable of no-interest in our analyses, in contrast to most previous studies which have failed to address this or provide information regarding the age distribution in each genotype subgroups. It should also be noted that we searched for differences between groups at each and every voxel using SPM analysis, in contrast to previous studies which have solely used different sets of predefined ROIs. However, the main difference between our study and previous series lies in the molecular characteristics of [^18^F]MPPF compared to [^11^C]WAY-100 635. Specifically, [^18^F]MPPF exhibits a relatively low affinity for 5-HT_1A _receptors, comparable to that of endogenous 5-HT, and appears to selectively bind to externalized receptors [8], whereas [^11^C]WAY-100 635 demonstrates greater affinity and a non-selective binding to both internalized and externalized receptors [35]. These differences may make [^18^F]MPPF more sensitive than [^11^C]WAY-100 635 to the extracellular concentration of 5-HT, as suggested in rodents [6]. In particular, [^18^F]MPPF BP_ND _might increase as a result of decreased 5-HT release [36]. Accordingly, the greater [^18^F]MPPF BP_ND _observed in healthy women homozygote carriers for the G allele, if confirmed, could reflect the downstream consequences of an increased expression of 5-HT_1A _autoreceptors in the raphe nuclei, i.e. a decreased release of 5-HT as well as a subsequent increased expression of post-synaptic 5-HT_1A _receptors. However, the lack of clear-cut changes in [^18^F]MPPF BP_ND _over the dorsal and median raphe nuclei does not support such a mechanism.

An original aspect of our study was to take into account both 5-HTTLPR and C(-1019)G 5-HT_1A _promoter polymorphisms, and to ensure that any effect of the latter on [^18^F]MPPF BP_ND _was not due to an imbalanced distribution of 5-HTTLPR alleles among the different groups considered. Interestingly, while both polymorphisms are associated with common features regarding [^18^F]MPPF BP_ND _(same gender specificity and greater [^18^F]MPPF BP_ND _in subjects with supposedly lower extracellular concentration of 5-HT), these were observed in distinct brain regions. Whereas differences in [^18^F]MPPF BP_ND _associated with 5-HTTLPR polymorphism were previously reported predominately over paralimbic regions (parahippocampal, orbitofrontal, insula, cingulate) [3], [^18^F]MPPF BP_ND _associated with C(-1019)G 5-HT_1A _promoter polymorphism, was primarily localised to the frontal and temporal neocortex in this study. These distinctive patterns might reflect the involvement of specific serotoninergic projection pathways.

Overall, our study failed to demonstrate any significant association between C(-1019)G 5-HT_1A _promoter polymorphism and regional distribution of [^18^F]MPPF BP_ND _in our entire sample. This negative finding was also observed in C/C and C/G women but our data suggest that the small number of women homozygote carriers for the G allele display greater [^18^F]MPPF BP_ND _relative to other individuals. Confirmation of this latter finding, together with the previous studies of 5-HTTLPR polymorphism, indicates that these two polymorphic regions are relevant co-variables of age and gender, which should be considered for future [^18^F]MPPF studies in normal individuals as well as patients.

## Competing interests

The authors declare that they have no competing interests.

## Authors' contributions

AL coordinated the study. FL participated to the execution of the study. CB carried out the genotype analyses. MA participated to the [^18^F]MPPF radiosynthesis. AL, NC and SB processed and analyzed the PET data. AL, NC, PG, and PR interpreted the results. AL, PG and PR drafted the manuscript. All authors read and approved the final manuscript.

## Supplementary Material

Additional file 1 **Plot of the [^18^F]MPPF BP_ND _values of 23 women at the peak voxel of the significant cluster in the left superior and middle frontal gyri (a) and in the right and left orbitofrontal cortex (b)**. the first six scans correspond to the women with C/C genotype, the scan number 7 to 21 represent the 15 women with C/G genotype and the two last scans represent the two women with G/G genotype. Click here for file

Additional file 2 **Plot of the [^18^F]MPPF BP_ND _values of 23 women at the peak voxel of the significant cluster in the right and left mesial frontal pole (a) and in the right second and third temporal cortex (b)**. The first six scans correspond to the women with C/C genotype, the scan number 7 to 21 represent the 15 women with C/G genotype and the two last scans represent the two women with G/G genotype. Click here for file
