# Supplementary figures and images for: 5-HT1A gene promoter polymorphism and [18F]MPPF binding potential in healthy subjects: a PET study
Source: Behav Brain Funct. 2010 Jul 7;6:37. doi: 10.1186/1744-9081-6-37 (PMC2909987; doi:10.1186/1744-9081-6-37)

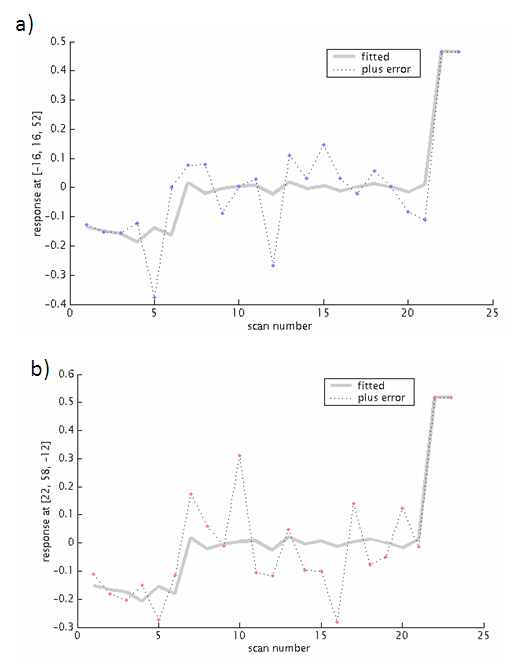

Supplement: Additional file 1 — Plot of the [18F]MPPF BPND values of 23 women at the peak voxel of the significant cluster in the left superior and middle frontal gyri (a) and in the right and left orbitofrontal cortex (b). the first six scans correspond to the women with C/C genotype, the scan number 7 to 21 represent the 15 women with C/G genotype and the two last scans represent the two women with G/G genotype. [file 1744-9081-6-37-S1.PNG]

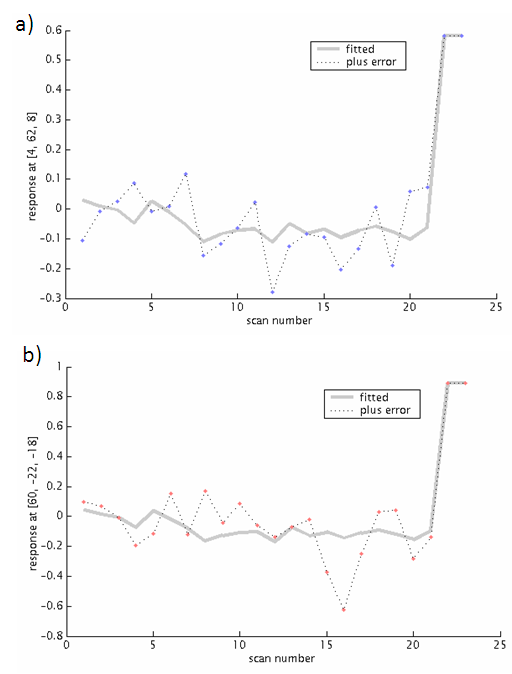

Supplement: Additional file 2 — Plot of the [18F]MPPF BPND values of 23 women at the peak voxel of the significant cluster in the right and left mesial frontal pole (a) and in the right second and third temporal cortex (b). The first six scans correspond to the women with C/C genotype, the scan number 7 to 21 represent the 15 women with C/G genotype and the two last scans represent the two women with G/G genotype. [file 1744-9081-6-37-S2.PNG]
